# One-leg rise performance and associated knee kinematics in ACL-deficient and ACL-reconstructed persons 23 years post-injury

**DOI:** 10.1186/s12891-019-2887-3

**Published:** 2019-10-25

**Authors:** Andrew Strong, Eva Tengman, Divya Srinivasan, Charlotte K. Häger

**Affiliations:** 1 Department of Community Medicine and Rehabilitation, Physiotherapy, Umeå University, SE-90187 Umeå, Sweden; 2 Department of Industrial and Systems Engineering, Virginia Polytechnic Institute and State University, Blacksburg, VA USA

**Keywords:** Knee injury, Knee control, Knee function, Lower limb, Osteoarthritis, Clinical assessment, Motion analysis, Biomechanics

## Abstract

**Background:**

Research indicates reduced knee function and stability decades after anterior cruciate ligament (ACL) injury. Assessment requires reliable functional tests that discriminate such outcomes from asymptomatic knees, while providing suitable loading for different populations. The One-leg rise (OLR) test is common in clinics and research but lacks scientific evidence for its implementation. Our cross-sectional study compared performance including knee kinematics of the OLR between ACL-injured persons in the very long term to controls and between legs within these groups, and assessed the within-session reliability of the kinematics.

**Methods:**

Seventy ACL-injured individuals (mean age 46.9 ± 5.4 years) treated with either reconstructive surgery and physiotherapy (ACL_R_; *n* = 33) or physiotherapy alone (ACL_PT_; *n* = 37), on average 23 years post-injury, and 33 age- and sex-matched controls (CTRL) attempted the OLR. Participants completed as many repetitions as possible to a maximum of 50 while recorded by motion capture. We compared between all groups and between legs within groups for total repetitions and decomposed the OLR into movement phases to compare phase completion times, maximum and range of knee abduction and adduction angles, and mediolateral knee control in up to 10 repetitions per participant.

**Results:**

ACL_PT_ performed significantly fewer OLR repetitions with their injured leg compared to the CTRL non-dominant leg (medians 15 and 32, respectively) and showed significantly greater knee abduction than ACL_R_ and CTRL (average 2.56°-3.69° depending on phase and leg). Distribution of repetitions differed between groups, revealing 59% of ACL_PT_ unable to complete more than 20 repetitions on their injured leg compared to 33% ACL_R_ and 36% CTRL for their injured and non-dominant leg, respectively. Within-session reliability of all kinematic variables for all groups and legs was high (ICC 3,*10* 0.97–1.00, 95% CI 0.95–1.00, SEM 0.93–1.95°).

**Conclusions:**

Negative outcomes of OLR performance, particularly among ACL_PT_, confirm the need to address aberrant knee function and stability even decades post-ACL injury. Knee kinematics derived from the OLR were reliable for asymptomatic and ACL-injured knees. Development of the OLR protocol and analysis methods may improve its discriminative ability in identifying reduced knee function and stability among a range of clinical populations.

## Background

Anterior cruciate ligament (ACL) injury often negatively affects knee function not only in the short term but also in the long term despite rehabilitative interventions [1]. Treatment involves either physiotherapy alone or in conjunction with additional reconstructive surgery, with conflicting evidence as to the best approach and a lack of informed guidance for individual tailoring. Regardless of treatment strategy, there is an elevated risk for re-injury/secondary injury during the subsequent years [2], as well as for longer term problems such as knee osteoarthritis (OA) [1]. Studies investigating the very long-term (> 20 years) effects of ACL injury on knee function are, however, scarce.

Clinically evaluating knee function following ACL injury often includes, for example, thigh muscle strength, knee range of motion, and jumping/hopping ability. The injured leg is commonly compared to the contralateral non-injured leg using the Limb Symmetry Index (LSI), where achieving > 90% of the outcome measures is a return-to-sport criteria [3]. However, the LSI has been shown to overestimate post-ACL injury quadriceps strength and hop ability [4, 5]. To complement the LSI, assessing movement quality during functional tests may reveal movement patterns that potentially predispose this group to further knee-related issues [6]. Reliable functional tests are therefore needed that can discriminate such patterns from asymptomatic knees while providing suitable loading for different populations, including those of an older age for longer term assessment.

The One-leg rise (OLR) test, involving standing and sitting from a stool with only 1 foot on the ground, has been applied in clinics and research to assess knee function. Thorstensson et al., (2004) found that chronic knee pain sufferers unable to perform 20 repetitions of the OLR were more likely to develop radiographic knee OA 5 years later [7]. The OLR was also more sensitive than gait at identifying changes in peak adduction moment following an exercise programme among the same cohort [8]. A one-leg test may be particularly advantageous when assessing ACL-injured individuals, who have been shown to reduce loading of the injured leg during double-leg squats [9]. In fact, worse knee confidence on average 9 years post-ACL reconstruction has been shown to be associated with poorer performance of the OLR [10]. Moreover, at 5–10 years post-ACL reconstruction, worse performance of the OLR has also been associated with greater tibiofemoral OA severity [11]. The OLR may thus be a relevant test of lower limb function among ACL-injured persons where both performance regarding the number of repetitions achieved and knee kinematics are of interest.

The potential added value of knee kinematics during the OLR would facilitate assessments of knee joint stability, defined here in accordance with Riemann and Lephart [12] as the ability to remain or promptly return to proper alignment, something that is believed to be a major contributing factor to long-term post-ACL injury knee problems such as OA [13]. Indeed, greater knee abduction of the injured leg compared to the non-injured leg during a one-leg half squat has been seen among non-operated ACL-injured males and females [14]. Greater knee abduction was also observed for the injured leg of non-operated ACL-injured persons compared to controls during tests such as a mini-squat, one-leg half squat and rising from half-kneeling [15]. Additionally, mediolateral knee control, as assessed by measures of knee position in the frontal plane, has been shown to be worse among ACL-injured persons compared to controls during a one-leg hop for distance [16]. Among ACL-injured males, poorer mediolateral knee control during a drop jump was associated with worse knee proprioception [17]. Thus, measures of mediolateral knee control during the OLR may provide additional valuable information regarding knee function among ACL-injured persons. However, a necessary first step before studying OLR knee kinematics to interpret knee function, is to assess the within-session reliability firstly among individuals with asymptomatic knees and secondly among the population of interest, something which we believe has not been done before.

Our aims in this study were to 1) assess the discriminative ability of OLR performance and knee kinematic outcome measures among ACL-injured persons, treated with and without surgical reconstruction, in the very long term after injury between the injured and non-injured legs and to controls without knee complaints, and 2) assess the within-session reliability of knee kinematics during execution of the OLR among asymptomatic individuals and ACL-injured cohorts. We hypothesised that both ACL-injured groups would show worse knee function and stability of the injured leg compared to the non-dominant leg of controls and to their non-injured contralateral leg, as characterised by significantly fewer OLR repetitions and greater knee abduction/adduction range of motion. We further hypothesised that the knee kinematics would show high within-session reliability.

## Methods

### Participants

This study forms part of the KACL20-study (Knee injury - Anterior Cruciate Ligament after more than 20 years), a cross-sectional research programme involving two ACL cohorts and a control group: 1) 33 ACL-injured persons treated with reconstructive surgery and physiotherapy (ACL_R_), 2) 37 ACL-injured persons treated with physiotherapy only (ACL_PT_), and 3) 33 age- and sex-matched controls (CTRL) with asymptomatic knees. ACL injuries occurred on average 23 (17–28) years prior to testing (see Table 1 and our previous article [18] for more details regarding demographics, background data, surgery techniques, physiotherapy treatment, and the recruitment process). ACL-injured participants were recruited from two different hospitals in Sweden. For ACL_R,_ physiotherapy treatment was provided for prehabilitation purposes for 3 months before reconstructive surgery was performed along with post-operative physiotherapy of at least 22 weeks. ACL_PT_ were treated solely with physiotherapy until specific screening tests could be performed sufficiently without instability or symptoms after a median time of 22 weeks (range 12–60 weeks). Exclusion criteria for the present study were bilateral ACL injury, other severe injury or disease to the non-injured leg, prosthesis, or any other musculoskeletal, rheumatological or neurological pathology. Controls were recruited through advertisement and convenience sampling and were matched to ACL-injured participants with regard to age and sex. In addition to self-reporting of asymptomatic knees, clinical examinations of controls were performed to exclude injury of the ACL, other ligaments, or the meniscus so that controls were deemed eligible for participation as controls in the study. All participants received prior information about the study before providing their written informed consent in accordance with the declaration of Helsinki. The study was approved by the Regional Ethical Review Board of Umeå, Sweden (Dnr. 08–211 M).

**Table 1:** Participant characteristics (mean (SD), unless otherwise stated)

	Groups		
	ACL_R_	ACL_PT_	CTRL
Participants (*n*)	33	37	33
Males/females (*n*)	21/12	23/14	21/12
Age at test (years)	45.6 (4.5)	48.1 (5.9)	46.7 (5.0)
Years since injury	23.9 (2.8)	23.1 (1.3)	–
Years between injury and surgery	3.6 (2.3)	–	–
Height (cm)	174.0 (9.1)	173.5 (8.0)	176.4 (9.8)
Weight (kg)	83.0 (15.6)	87.1 (14.9)	77.4 (14.9)^a^
BMI (kg/m^2^)	27.2 (3.3)	28.9 (4.6)	24.6 (2.5)^b^
Injury side: dominant/non-dominant	21/12	20/17	–
*Cause of injury:*			
Soccer (*n*)	24	25	–
Alpine skiing (*n*)	2	5	–
Other sports (*n*)	6	2	–
Non-sport related (*n*)	1	5	–
OA K&L^c^ 1 (*n*)	5	6	–
OA K&L 2 (*n*)	12	13	–
OA K&L 3 (*n*)	10	9	–
OA K&L 4 (*n*)	4	3	–

Table adapted from Tengman et al. (15)

*Abbreviations*: *BMI* Body mass index, *OA* Osteoarthritis, *SD* Standard deviation

Significant differences:

^a^ACL_PT_ vs. CTRL, *p* = 0.025

^b^ACL_R_ vs. CTRL, *p* = 0.014; ACL_PT_ vs. CTRL, *p* < 0.001

^c^Radiographic OA was graded according to Kellgren & Lawrence [19]; K&L 0–1 = no-or-low, K&L 2–4 = moderate-to-high degree of OA

### Procedures and data collection

The OLR was performed as part of a test battery consisting of nine different tests at the U-Motion laboratory, Umeå University, Sweden. Participants began the OLR by sitting on a stool (height 0.48 m) and were asked to perform as many repetitive sit-stand-sit movements as possible at a self-selected but controlled speed with only 1 foot on the floor and arms across the chest. Participants continued to either failure or were stopped if achieving 50 repetitions in line with the protocol by Hart et al., [10], although they were unaware of this maximum prior to the test. Failure was defined if the contralateral non-weight bearing leg/foot contacted the weight-bearing leg or the ground or if the foot of the standing leg moved position. ACL-injured participants started with their non-injured leg and controls with their dominant leg (dominance defined as the leg preferred to kick a ball) after performing one practice repetition. The test was repeated with the contralateral leg after a minimum two-minute rest. An eight-camera three-dimensional motion capture system (Oqus Qualisys, Gothenburg, Sweden, 240 Hz) and one two-dimensional video camera recorded all movements. Qualisys Track Manager software (version 2.2, Qualisys, Gothenburg, Sweden) was used to capture and track 42 retro-reflective markers which were affixed by double-sided adhesive tape to the skin of the participants on specific anatomical landmarks of the trunk and lower body according to an adapted Helen Hayes marker set, described in more detail in our previous article [20].

### Data analysis

#### Performance screening and analysis

The performance outcome variable of the OLR test was defined as the total number of consecutive successful repetitions achieved for each respective leg. In this respect, one repetition of the OLR was defined as beginning when participants were no longer in contact with the stool and ended once participants next sat on the stool after having achieved a standing position, defined and controlled visually by the test leader during testing as a fully extended knee, on the tested leg. All repetitions were checked for adherence to the desired protocol firstly by the lead author using video footage and when uncertainty arose together with a co-author (ET). Unsuccessful trials, along with subsequent attempts, were omitted from all analyses. However, when participants performed the first repetition incorrectly and continued with successful attempts, the first repetition was omitted but subsequent successful repetitions were included.

#### Data processing and reduction

**Fig. 1:**
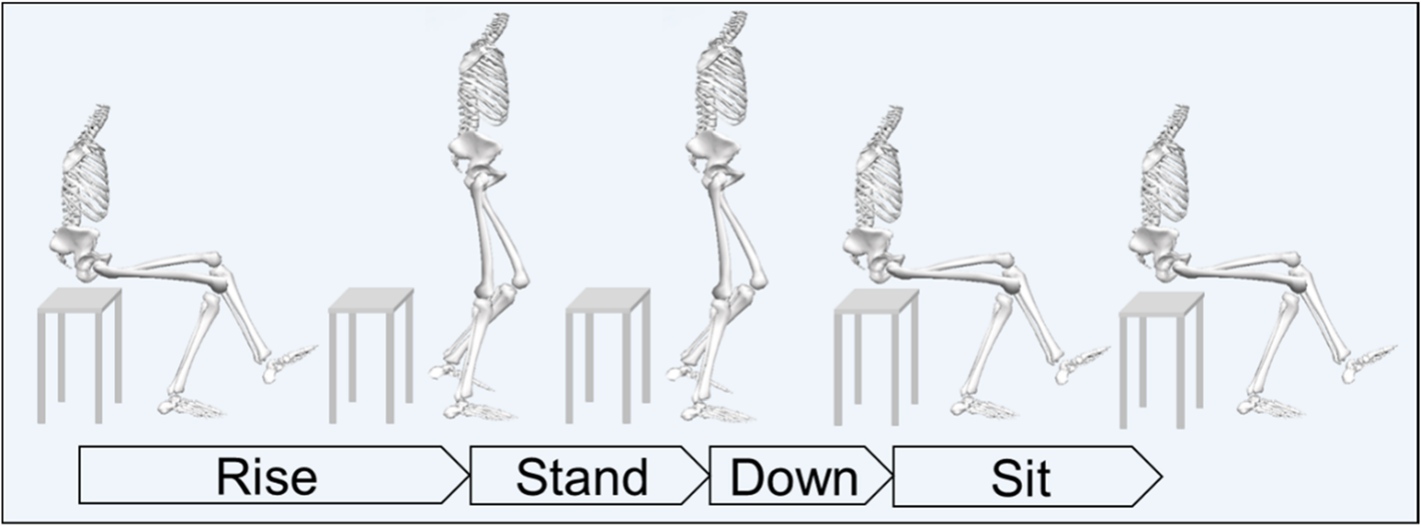
A schematic illustration of the four defined phases for one repetition of the One-leg rise test. (Fig. 1 was conceived, designed and produced by the authors of this manuscript)

Marker trajectory data were gap-filled using polynomial interpolation in Qualisys Track Manager software when deemed accurate up to a maximum of 10 frames per sequence. Marker data were then exported to Visual3D software (Visual3D Professional version 5.02.23, C-Motion Inc., Germantown, Maryland, USA) and low-pass filtered using a second order Butterworth filter with a cut-off frequency of 6 Hz. A five-segment rigid-body model consisting of two shanks, two thighs and one pelvis was then constructed, with joint centres based on a 6-degrees-of-freedom model. Knee joint angles were defined as the rotation of the shank relative to the thigh using the Cardan X-Y-Z convention, so that (with positive rotations from zero presented first, i.e. positive/negative) X represented flexion/extension, Y represented adduction/abduction, and Z represented internal/external rotation [21]. We decomposed the OLR into four phases (Fig. 1) with start and end times determined by the vertical velocity of the hip joint centre, where positive values equate to an upwards direction. Maximum and minimum velocity were identified for each included repetition of each participant. Phases were thus defined as follows: i) *Rise* began when the hip joint centre velocity first exceeded 10% of its maximum, ii) *Stand* began when the hip joint centre velocity was next below 10% of its maximum, iii) *Down* began when the hip joint centre velocity was next below 10% of its minimum, and iv) *Sit* began when the hip joint centre velocity next exceeded 10% of its minimum. All events were checked manually and adjusted if considered incorrect.

#### Kinematic analyses

To capture representative knee kinematics during the OLR, the first successful repetition was omitted from kinematic analyses in line with a previous study [8] in order to avoid adjustments of body position. Thus, all consecutive successful repetitions from attempts 2 to 11 for each participant were analysed, resulting in up to 10 repetitions per participant. Ten repetitions were chosen to gain a reasonably representative analysis of movement patterns. Including more than 10 repetitions however, may have increased fatigue effects which have been shown to effect knee kinematics among ACL-reconstructed women [22]. We calculated the percentage time taken to complete each of the four movement phases within each repetition. For each *Rise* and *Down* phase we calculated maximum knee abduction and adduction angles, knee abduction/adduction range of motion, and mediolateral knee control. Mediolateral knee control was investigated by calculating the total number of knee movement units, based on a measure commonly used to assess arm reaching movements [23]. We operationally defined knee movement units as each knee velocity peak in the frontal plane on the knee velocity profile > 10% of the maximum knee velocity in the same direction. Means and standard deviations (SD) for maximum knee abduction, adduction, abduction/adduction range, and total movement units were subsequently calculated for the *Rise* and *Down* phases of all included repetitions for each individual.

#### Statistical analyses

Statistics were performed using IBM SPSS Statistics for Windows, version 23.0 (IBM Corp., Armonk, N.Y., USA). Outliers in the kinematic data (2.93% of the total data set) were reviewed and corresponding repetitions were cross-checked using video and motion capture recordings. No outliers were deemed to be due to technical or data entry errors and therefore none were removed from the statistical analyses. Skewness and Shapiro-Wilk tests were performed to assess data distributions. Subsequently, between-group comparisons of demographics for age, height, weight, and body mass index (BMI) were performed using One-way analysis of variance (ANOVA) tests due to normal data distributions and significant results followed up with Bonferroni post hoc tests. Between-group analyses of performance (number of repetitions and phase completion time) and knee kinematics compared ACL-injured legs to each other and to the non-dominant legs of CTRL as well as the non-injured legs of the ACL-injured groups to each other and to the dominant legs of CTRL, for a stringent comparison. Bland-Altman plots for kinematic variables were used to screen for systematic bias between repetition 2 and 11 [24]. All between-group performance and kinematic variables, including the LSI which was calculated for each individual by dividing the outcome measure for the injured or non-dominant leg by that of the non-injured or dominant leg respectively and multiplying by 100, were analysed using non-parametric Kruskal Wallis tests due to non-normally distributed data and significant results were followed up with Dunn-Bonferroni post hoc pairwise tests. Estimates of the effect sizes (*r*) for significant between group comparisons were calculated using the Z statistic of Mann-Whitney U tests:

$$ r=\frac{\mathrm{Z}}{\surd n} $$

where Z = Mann-Whitney U Z statistic.

*n* = the number of participants.

Cumulative percentages of completed repetitions were calculated and these distributions were statistically compared between groups using two-sample Kolmogorov-Smirnov tests. The total number of participants who completed 20 repetitions was compared between groups with Pearson’s chi-squared test. Within-group comparisons compared between legs within each group using Wilcoxon Signed Ranks tests and the associated Z statistic. Estimates of the effect sizes (*r*) for significant within-group comparisons were calculated using the Z statistic [25]:

$$ r=\frac{\mathrm{Z}}{\surd n} $$

where Z = Wilcoxon Signed Ranks Z statistic.

*n* = the number of pairs.

Effect sizes were considered large if 0.5, medium if 0.3 and small if 0.1 [25]. Significance levels were set a priori (α = 0.05). Within-session reliability was calculated for the knee kinematics of all groups and legs based on repetitions 2–11. Reliability was calculated for the following variables of the knee separately for both the *Rise* and *Down* phases: 1) maximum abduction, 2) maximum adduction, 3) maximum abduction/adduction range, and 4) movement units in the frontal plane. Intraclass Correlation Coefficient for a two-way mixed model where the mean of repeated measures (ICC 3,k) and absolute agreement was calculated [26].

ICC classification of reliability was made according to Fleiss [27], thus ICC < 0.40 = poor, ICC > 0.40 but < 0.75 = fair to good, and ICC > 0.75 = excellent. The standard error of measurement (SEM) was calculated to provide an estimate of the error in the units of measurement, thus giving clinically relevant values for expected error in each individual. It was calculated as the square root of the mean square error term from the ANOVA [28].

## Results

### Performance of the OLR test

#### Total repetitions

**Fig. 2:**
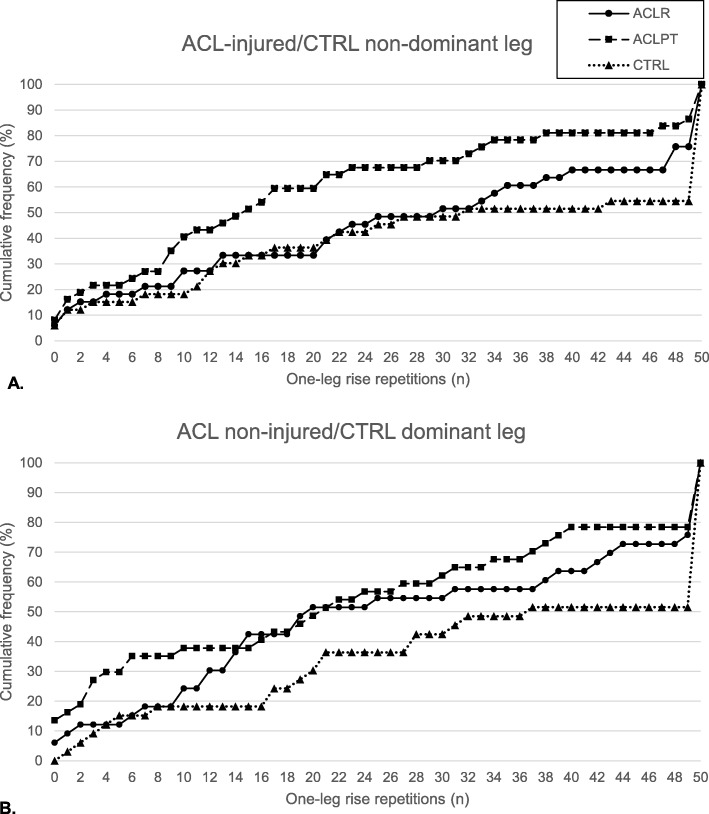
Graphs displaying the amount of repetitions completed by all participants from each group as cumulative percentages clearly demonstrating the difference in distribution between groups. A) the non-injured leg of ACL_R_ and ACL_PT_ and the dominant leg of CTRL, and B) the injured leg of ACL_R_ and ACL_PT_ and the non-dominant leg of CTRL

All participants were included in performance analyses for total repetitions. The number of participants completing the maximum 50 repetitions were for the ACL-injured/CTRL non-dominant leg: *n* = ACL_R_ 8, ACL_PT_ 5, CTRL 12, and for the ACL non-injured/CTRL dominant leg: *n* = ACL_R_ 8, ACL_PT_ 8, CTRL 16. Median (Quartile 1, Quartile 3) successful repetitions for the ACL-injured/CTRL non-dominant leg were: ACL_R_ 30 (10, 49), ACL_PT_ 15 (6.5, 33.5), CTRL 32 (12, 50), and for the ACL non-injured/CTRL dominant leg: ACL_R_ 20 (11, 49.5), ACL_PT_ 21 (3, 39.5), and CTRL 37 (18, 50). ACL_PT_ performed significantly fewer repetitions than CTRL for the injured leg compared to the non-dominant leg respectively (*r =* − 0.27, *p* = 0.050). Distributions of cumulative percentages are displayed in Fig. 2 and were significantly different (*p* < 0.02) for all between group comparisons except for between the non-injured leg of ACL_R_ and ACL_PT_. For the ACL-injured/CTRL non-dominant leg comparisons, 59% of ACL_PT_ were unable to complete 20 repetitions, compared to 33% ACL_R_ and 36% CTRL, although these between-group differences were not significant. No other between- or within-group differences were statistically significant for total repetitions. The number of participants unable to perform any repetitions for the ACL-injured/ CTRL non-dominant leg were: *n* = ACL_R_ 2, ACL_PT_ 3, CTRL 2, and for the ACL non-injured/CTRL dominant leg: *n* = ACL_R_ 2, ACL_PT_ 5, CTRL 0. The LSI was calculated for each individual who had performed at least one repetition on each leg (*n* = ACL_R_ 31, ACL_PT_ 32, CTRL 31) but was not significantly different between groups (medians (Quartile 1, Quartile 3)): ACL_R_ 100 (90.5, 157.1), ACL_PT_ 100 (57.9, 104.8), CTRL 100 (67.7, 100)).

#### Time of completion – phase percent time

Time of completion analyses, as well as for knee kinematics, required participants to complete more than one repetition of the OLR. Therefore, participants included in between-group comparisons were: ACL-injured/CTRL non-dominant leg, *n* = ACL_R_ 29, ACL_PT_ 31, CTRL 29; ACL non-injured/CTRL dominant leg, *n* = ACL_R_ 30, ACL_PT_ 31, CTRL 32. No between-group differences were statistically significant for percent time of phase completion (Table 2). Within-group (between-leg) analyses for time completion, and for knee kinematics, required each participant to complete more than one repetition on both legs. As such, the number of participants included in this analysis was further reduced for ACL_PT_ and thus included participants for within-group comparisons were: *n* = ACL_R_ 29, ACL_PT_ 28, CTRL 29. ACL_PT_ spent significantly longer in the *Sit* phase when using their non-injured leg compared to their injured leg (median difference 0.12 s, Z = 2.18, *r* = 0.41, *p* = 0.03). CTRL took significantly longer to complete the *Rise* phase with their non-dominant leg compared to their dominant leg (median difference 0.62%, Z = − 2.53, *r* = − 0.45, *p* = 0.01), but significantly longer to complete the Down phase with their dominant leg compared to their non-dominant leg (median difference 0.03 s, Z = 2.07, *r* = 0.38, *p* = 0.04). All significant differences were thus of medium effect size and no other within-group differences were statistically significant for percent time of phase completion.

**Table 2:** Comparisons of normalised time (%) and total time (sec) of completion for each phase of the One-leg-rise test for all groups and both legs. Values are group medians (Quartile 1, Quartile 3) related to between-group comparisons. Between-leg comparisons within groups were based on differences between legs of each individual

*Phases and legs*	Normalised time (%)			Total time (sec)		
	ACL_R_	ACL_PT_	CTRL	ACL_R_	ACL_PT_	CTRL
*Rise*						
Inj/ND	27.94 (25.50, 33.00)	27.66 (25.71, 31.96)	**29.94 (26.83, 33.86)** ^a^	0.99 (0.87, 1.21)	0.96 (0.87, 1.06)	0.96 (0.81, 1.10)
NI/Dom	28.39 (23.94, 31.79)	28.15 (26.16, 31.30)	**28.27 (25.05, 30.39)** ^a^	1.04 (0.89, 1.15)	0.95 (0.86, 1.12)	0.93 (0.85, 1.14)
*Stand*						
Inj/ND	13.13 (10.10, 19.80)	14.57 (10.03, 23.96)	13.41 (8.69, 18.37)	0.42 (0.30, 0.84)	0.50 (0.28, 0.86)	0.40 (0.24, 0.59)
NI/Dom	14.77 (11.15, 20.11)	13.99 (9.89, 18.97)	13.33 (8.73, 15.62)	0.53 (0.36, 0.91)	0.48 (0.28, 0.69)	0.44 (0.29, 0.58)
*Down*						
Inj/ND	31.13 (24.96, 36.02)	32.93 (27.97, 37.37)	30.68 (28.31, 36.23)	1.03 (0.95, 1.31)	1.08 (0.95, 1.28)	**0.99 (0.88, 1.19)** ^c^
NI/Dom	31.67 (24.96, 34.52)	31.11 (29.14, 34.17)	30.56 (26.73, 33.02)	1.07 (0.98, 1.27)	1.08 (0.91, 1.32)	**1.02 (0.93, 1.17)** ^c^
*Sit*						
Inj/ND	29.48 (23.28, 33.57)	24.39 (17.75, 29.11)	27.43 (24.88, 32.04)	0.97 (0.75, 1.31)	**0.78 (0.65, 1.02)** ^b^	0.91 (0.68, 1.17)
NI/Dom	27.08 (18.58, 32.52)	27.33 (20.01, 30.39)	28.80 (25.42, 33.20)	0.88 (0.64, 1.41)	**0.90 (0.66, 1.10)** ^b^	1.02 (0.87, 1.21)

Bold text highlights significant differences

*Abbreviations*: *Inj* ACL-injured, *ND* CTRL non-dominant, *NI* ACL non-injured, *Dom* CTRL dominant

Significant within-group (between-leg) differences:

^a^CTRL *Rise* phase normalized time; dominant leg vs. non-dominant leg (Z = − 2.53, *r* = − 0.45, *p* = 0.01)

^b^ACL_PT_ *Sit* phase total time; injured leg vs. non-injured leg (Z = 2.18, *r* = 0.41, *p* = 0.03)

^c^CTRL *Down* phase total time; dominant leg vs. non-dominant leg (Z = 2.07, *r* = 0.38, *p* = 0.04).

### Knee kinematic variables

**Fig. 3:**
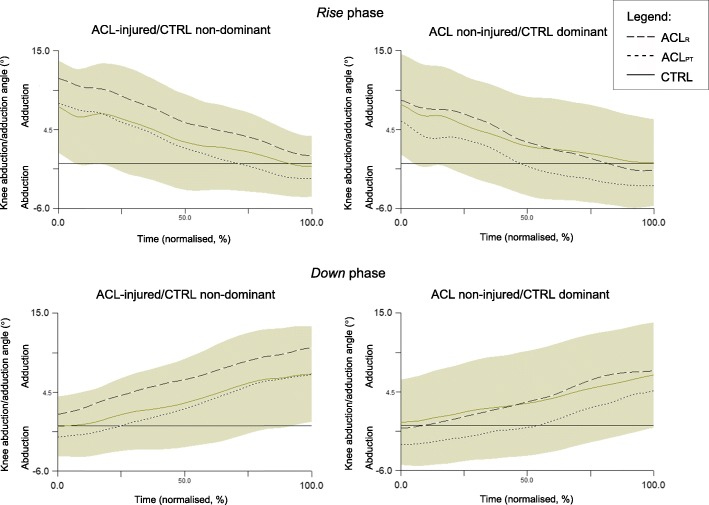
Mean knee abduction/adduction angles (°) for all groups throughout the *Rise* and *Down* phases of the One-leg rise for both legs for up to 10 repetitions per participant. The shaded area represents the standard deviation for the control group. Positive angles indicate knee adduction and negative angles indicate knee abduction. Time has been normalised and is provided in percent

Included participants for kinematic analyses are stated above in the section *Time of completion – phase percent time*. Fig. 3 shows mean knee angle curves in the frontal plane for all groups throughout the *Rise* and *Down* phases for both legs in up to 10 repetitions per participant. During the *Rise* phase, ACL_PT_ displayed on average 2.6° greater knee abduction maximum than ACL_R_ for their injured leg (*r* = − 0.33, *p* = 0.038) and 3.4° greater for their non-injured leg (*r* = − 0.36, *p* = 0.034) as well as 3.6°greater for their non-injured leg compared to the dominant leg of CTRL (*r* = − 0.32, *p* = 0.021) (Table 3). During the *Down* phase, ACL_PT_ displayed on average 3.7° greater knee abduction maximum for their injured leg compared to the injured leg of ACL_R_ (*r* = − 0.32, *p* = 0.029) and 3.1° greater for the non-injured leg compared to the dominant leg of CTRL (*r* = − 0.31, *p* = 0.036). No between-group differences were statistically significant for knee adduction maximum, knee adduction/abduction range or knee movement units (Table 3). ACL_R_ displayed on average 1.04° greater knee abduction maximum in their non-injured leg compared to their injured leg during the *Down* phase (Z = − 2.11, *r* = − 0.39, *p* = 0.035). Further, ACL_R_ displayed on average 1.95° greater knee adduction maximum in their injured leg than their non-injured leg during the *Down* phase (Z = − 2.04, *r* = − 0.38, *p* = 0.041). No within-group differences were evident for knee abduction/adduction range or knee movement units (see Table 3).

**Table 3:** Between-group comparisons based on group medians (Quartile 1, Quartile 3) for knee abduction maximum, knee adduction maximum, knee abduction/adduction range, and knee movement units in the frontal plane during the *Rise* and *Down* phases of the One-leg rise. *P*-values (*p*) and effect sizes (r) are provided for all significant results

*Variables*	Groups			Group comparisons		
	ACL_R_	ACL_PT_	CTRL	ACL_R_-ACL_PT_	ACL_R_-CTRL	ACL_PT_-CTRL
*Rise* phase						
Knee abd max (°)						
Inj/ND	**0.51 (2.47, −2.67)**	**3.07 (7.47, −0.35)**	0.68 (5.00, −1.55)	*p* = 0.038, *r* = − 0.33	NS	NS
NI/Dom	**1.67 (4.85, −1.08)**	**5.03 (6.52, 2.69)**	**1.40 (6.25, −3.13)**	*p* = 0.034, *r* = − 0.36	NS	*p* = 0.021, *r* = − 0.32
Knee add max (°)						
Inj/ND	10.14 (7.49, 15,67)	7.94 (3.85, 15.45)	8.27 (4.29, 12.89)	NS	NS	NS
NI/Dom	8.99 (6.74, 12.17)	6.51 (0.95, 9.82)	6.82 (2.96, 14.35)	NS	NS	NS
Knee abd/add range (°)						
Inj/ND	11.40 (7.74, 14.57)	12.30 (7.48, 15.87)	10.54 (7.27, 12.93)	NS	NS	NS
NI/Dom	11.00 (8.54, 13.33)	10.49 (8.08, 15.16)	8.85 (6.94, 12.10)	NS	NS	NS
Knee MU (n)						
Inj/ND	4.67 (4.35, 5.75)	5.00 (4.17, 5.33)	5.00 (4.30, 5.70)	NS	NS	NS
NI/Dom	5.20 (4.68, 6.00)	5.10 (4.22, 5.45)	4.60 (4.20, 5.30)	NS	NS	NS
*Down* phase						
Knee abd max (°)						
Inj/ND	**−0.56 (2.20, −4.24)** ^a^	**3.13 (6.65, −1.39)**	0.67 (5.06, − 1.70)	*p* = 0.029, *r* = − 0.32	NS	NS
NI/Dom	**0.48 (4.64, −2.23)** ^a^	**4.04 (6.43, 2.41)**	**0.96 (5.30, −3.68)**	NS	NS	*p* = 0.036, *r* = − 0.31
Knee add max (°)						
Inj/ND	**9.24 (6.52, 15.42)** ^**b**^	8.03 (2.95, 13.04)	9.25 (3.84, 11.83)	NS	NS	NS
NI/Dom	**7.29 (5.48, 11.95)** ^**b**^	4.80 (−0.06, 8.95)	5.78 (1.91, 13.81)	NS	NS	NS
Knee abd/add range (°)						
Inj/ND	8.90 (6.41, 13.50)	9.57 (6.73, 14.30)	8.57 (6.09, 10.76)	NS	NS	NS
NI/Dom	8.08 (5.97, 12.18)	9.60 (5.80, 12.25)	7.16 (5.58, 10.57)	NS	NS	NS
Knee MU (n)						
Inj/ND	4.90 (3.95, 5.70)	5.50 (4.70, 6.25)	4.60 (3.90, 5.60)	NS	NS	NS
NI/Dom	4.80 (4.28, 5.65)	5.15 (4.28, 6.10)	4.70 (4.08, 5.35)	NS	NS	NS

Bold text highlights significant differences

*Abbreviations*: *abd* Abduction, *add* Adduction; *Inj* ACL-injured, *ND* CTRL non-dominant, *NI* ACL non-injured, *Dom* CTRL dominant, *NS* Not statistically significant, *MU* Movement units

Significant within-group (between-leg) differences,

^a^Knee abduction maximum for ACL_R_ during *Down* phase; injured leg vs. non-injured leg, *p* = 0.035, *r* = − 0.39

^b^Knee adduction maximum for ACL_R_ during *Down* phase; injured leg vs. non-injured leg, *p* = 0.041, *r* = − 0.38

### Within-session reliability of knee kinematics

All knee kinematic variables showed excellent within-session reliability for all groups and legs (ICC (3,*10*) 0.81–1.00, 95% CI 0.67–1.00, SEM 0.93–1.95) during both the *Rise* and *Down* phase (Table 4).

**Table 4:** Within-session reliability of the knee kinematic variables during One-leg rise test performance for all groups

*Variables*	Groups								
	ACL_R_			ACL_PT_			CTRL		
*Rise* phase									
	ICC (3,k)	95% CI	SEM	ICC (3,k)	95% CI	SEM	ICC (3,k)	95% CI	SEM
Knee abd max (°)									
Inj/ND	0.99	0.99–1.00	1.03	0.99	0.99–1.00	1.21	1.00	0.99–1.00	1.04
NI/Dom	0.99	0.99–1.00	1.04	0.99	0.99–1.00	1.17	0.99	0.99–1.00	1.37
Knee add max (°)									
Inj/ND	1.00	0.99–1.00	1.45	1.00	0.99–1.00	1.57	0.99	0.99–1.00	1.30
NI/Dom	0.99	0.98–1.00	1.45	1.00	0.99–1.00	1.31	1.00	0.99–1.00	1.04
Knee abd/add range (°)									
Inj/ND	0.99	0.98–1.00	1.86	0.99	0.98–1.00	1.85	0.98	0.96–0.99	1.57
NI/Dom	0.99	0.98–0.99	1.69	0.99	0.98–1.00	1.64	0.97	0.95–0.99	1.59
Knee MU (*n*)									
Inj/ND	0.86	0.76–0.93	1.05	0.93	0.88–0.97	1.24	0.87	0.78–0.93	1.06
NI/Dom	0.81	0.67–0.91	1.13	0.86	0.74–0.93	0.98	0.86	0.76–0.93	1.05
*Down* phase									
	ICC (3,k)	95% CI	SEM	ICC (3,k)	95% CI	SEM	ICC (3,k)	95% CI	SEM
Knee abd max (°)									
Inj/ND	1.00	0.99–1.00	0.93	0.99	0.99–1.00	1.46	0.99	0.99–1.00	1.06
NI/Dom	0.99	0.99–1.00	0.93	1.00	0.99–1.00	0.93	1.00	0.99–1.00	1.29
Knee add max (°)									
Inj/ND	0.99	0.99–1.00	1.60	1.00	0.99–1.00	1.57	0.99	0.99–1.00	1.44
NI/Dom	0.99	0.98–1.00	1.51	1.00	0.99–1.00	1.34	1.00	0.99–1.00	0.99
Knee abd/add range (°)									
Inj/ND	0.99	0.98–1.00	1.83	0.98	0.97–0.99	1.95	0.98	0.97–0.99	1.59
NI/Dom	0.99	0.98–1.00	1.71	0.99	0.98–0.99	1.62	0.98	0.97–0.99	1.52
Knee MU (*n*)									
Inj/ND	0.87	0.78–0.94	1.15	0.90	0.82–0.95	1.43	0.92	0.87–0.96	1.10
NI/Dom	0.87	0.78–0.94	1.29	0.85	0.74–0.93	1.21	0.89	0.81–0.94	1.38

Included participants were those who provided kinematic data for 10 repetitions for each respective leg, *n* = ACL_R_ Inj 23; ACL_R_ NI 23; ACL_PT_ Inj 22; ACL_PT_ NI 22; CTRL ND 27; CTRL Dom 25

*Abbreviations*: *ICC* Intraclass correlation coefficient, *CI* 95% confidence intervals, *SEM* Standard error of measurement, *abd* Abduction, *add* Adduction, *max* Maximum, *Inj* ACL-injured leg, *ND* CTRL non-dominant leg, *NI* ACL non-injured leg, *Dom* CTRL dominant leg, *MU* Movement units

## Discussion

ACL-injured persons treated solely with physiotherapy performed significantly fewer OLR repetitions than age- and sex-matched persons with asymptomatic knees when using their injured and non-dominant leg respectively, albeit with a small effect size. The distribution of cumulative repetitions for ACL-injured/CTRL non-dominant leg comparisons revealed that 59% of ACL_PT_ were unable to achieve the 20-repetition cut-off for predicting knee OA development stated by Thorstensson et al., (2004) compared to 33% ACL_R_ and 36% CTRL, although these differences were not statistically significant. ACL_PT_ also displayed significantly greater knee abduction of medium effect sizes than both ACL_R_ and CTRL during the *Rise* and *Down* phases of the OLR. Despite this, our findings showed inconsistent differences, particularly of knee kinematics, when comparing the ACL-injured groups to CTRL. This contradicted our previous research which found negative outcomes for the same ACL groups when compared to CTRL with regard to reduced control of single-limb stance [29], lower self-reported knee function and hop/jump capacity [18], and reduced knee muscle strength [30], as well as altered movement patterns during hop tests [20, 31, 32]. Thus the knee kinematics during the OLR, as performed and analysed in our study, did not discriminate certain existing disparities in knee movement control in the very long term after ACL injury.

Nevertheless, there was greater maximal knee abduction among ACL_PT_ compared to ACL_R_ and CTRL, although the differences were rather small but still significantly different. These differences in knee abduction align with a previous study of the same groups during landings from one-leg hops [20], although the clinical relevance in relation to detectable change remains to be determined. This finding is however further supported by Zhang and colleagues [33] who found greater knee abduction among ACL-deficient persons on average 5 years after injury compared to controls at heel contact during gait. Trulsson et al., [15] observed a greater medial position of the knee relative to the foot among non-operated ACL-injured persons compared to controls when performing a battery of tests including a mini-squat. That said, a more medial position of the knee would not necessarily result in knee abduction, which is more specifically defined by rotation of the shank relative to the thigh. The greater knee abduction for the non-injured leg of ACL_R_ in our study compared to their injured leg during the *Down* phase indicates even bilateral effects of the ACL injury. Indeed, reduced balance during a single-leg stance for both legs was previously seen for our ACL_R_ and ACL_PT_ groups [29]. Culvenor et al., [34] also reported reduced postural control 12 months post-ACL_R_ for both legs when performing single-leg squats. One possible explanation for these bilateral effects may be neuroplastic changes following ACL injury, of which there is growing evidence [35, 36].

Advantages of the OLR include its convenience due to the lack of required equipment or space. The consistent stool height (0.48 m) used in our study is similar to that which is encountered daily and enhances ecological validity. The movement itself resembles the everyday task of standing and sitting which can provide a relevant evaluation of an individual’s independence while isolating performance between legs. However, despite requiring more muscular effort than two-legged closed kinetic chain exercises, the OLR has been shown not to produce greater strains on the ACL than such tasks and can be considered appropriate for ACL-injured persons who can perform, for example, a traditional two-legged squat [37]. Further, the relative simplicity of the OLR compared to, e.g. a one-leg hop for distance, improves feasibility among populations of different ages and conditions. Nevertheless, the OLR requires adequate lower limb strength and endurance, coordination, balance and proprioceptive ability, factors which deteriorate across the life span. The OLR thus encompasses a number of important outcome variables for assessment of movement control. Furthermore, within-session reliability of our knee kinematic variables was excellent for all groups and legs, thus indicating that the observed movement patterns of these groups are consistent during repetitions 2–11 of the OLR and that averaged values are likely representative of each individual. This was also supported by the lack of systematic bias seen in Bland-Altman plots for these variables. Our proposal for assessing mediolateral knee control based on knee movement units revealed neither between- nor within-group differences for our comparisons in the present task. A similar movement control measure of the knee denominated fluency, defined as the number of times the velocity of the knee position in the coronal plane crossed zero when averaged per second, has however revealed worse mediolateral knee control among ACL-injured persons compared to controls during a one-leg hop for distance [16]. It is thus possible that our measure of knee movement units may discriminate movement control disparities in other more demanding tests and among populations with more severe pathologies and warrants further investigation.

Limitations of our study include the maximum 50 repetitions, applied to reduce fatigue effects on between-leg comparisons as well as the extreme delayed onset muscle soreness evident during pilot testing with no maximum. Statistically this created a ceiling effect and results would likely have been different without this maximum considering that 34 of 106 participants completed 50 repetitions on at least one leg and that CTRL accounted for 16 of those. Further, up to 229 repetitions were achieved in a previous study of chronic knee pain sufferers of similar age [7]. Additionally, the LSI was not an appropriate measure due to the maximum repetition limit and for those unable to perform a repetition on at least one leg. The determination of leg dominance, used to provide the most stringent comparison to controls by comparing the hypothesised less-competent and more competent legs separately between groups, i.e. ACL-injured vs. CTRL non-dominant and vice versa, was made according to which leg the participants preferred to kick a ball. However, recent evidence shows that certain healthy individuals change leg preference depending on the task involved [38], which may also be true for the OLR and for some injured persons. Thus, whether or not our between-group analysis resulted in the most stringent comparisons regarding injury side and dominance remains unclear. Our cross-sectional study design with long-term follow-up means that treatment strategies for ACL injuries have evolved since our participants were injured. Thus, our specific results may not be relevant for all ACL-injured persons. Other confounding factors across the two decades since injury such as, e.g. physical activity level, are also likely to have affected the outcome measures. We used 10% of the maximum/minimum hip joint centre velocity as a threshold level for setting the start/stop events of the OLR phases. Due to the lack of previous research investigating OLR kinematics, this decision was based on our own testing of various threshold levels across a number of participants and repetitions. Although we deemed this threshold level to be more appropriate than the alternatives that we tested, it is possible that choosing another threshold level may have changed the outcome of the results and thus further research is required to establish the most appropriate method. Further, there are common technical limitations to three-dimensional analyses, such as visibility of markers (hip and foot markers were often obscured when participants leaned forwards and due to the stool, respectively) or soft tissue artefacts which we tried to minimize using cluster markers and placement on solid anatomical landmarks [39]. The use of maximum values for kinematic variables is also sensitive to such artefacts and thus as well as data filtering, thorough manual checks were performed on movement profiles and data values in an attempt to ensure representative data.

Our study is the first to evaluate reliability of knee kinematics during execution of the OLR as well as implement the test to compare between legs of ACL-injured persons in the very long term after injury and to controls with asymptomatic knees. In future, adjustments to our protocol may help to improve the standardisation and discriminative ability of the OLR, which may lead to more successful application within research and clinics. Removing the maximum repetition limit, for example, appears feasible and should benefit interpretation. However, this may take a rather long time for completion, depending on the patient’s functional state, which may make it less feasible for application in clinical settings. Further, standardisation regarding performance speed, e.g. using a metronome, may be considered. Randomisation of leg order in research studies would also help to avoid potential fatigue bias. The addition of kinetic data to enable analysis of body centre of pressure and joint moments is likely to provide valuable biomechanical information. Although advanced three-dimensional analysis was used in this study, if specific key movement control outcome variables can be identified, the use of simpler and less expensive video and software solutions may add value to clinical implementation of the OLR. Further reliability analysis should establish the minimum number of OLR repetitions required to provide reliable knee kinematic data, fatigue effects and additional pathological groups.

## Conclusions

As long as two decades after injury, ACL-injured persons treated solely with physiotherapy performed fewer OLR repetitions than age- and sex-matched persons with asymptomatic knees when comparing the injured to the non-dominant leg respectively. The OLR also revealed greater knee abduction angles for ACL_PT_ compared to ACL_R_ and CTRL, indicating residual abnormal lower limb movement patterns. These results should however be interpreted with caution with regards to the potential treatment effects due to the very long time since injury and because this was not a randomised controlled study. The within-session reliability of the knee kinematics during the OLR among asymptomatic and ACL-injured knees was excellent and thus these measures are worth further exploration for use in research and clinics. Development of the OLR protocol and analysis methods may further improve its discriminative ability in identifying reduced knee function and abnormal movement patterns in research and clinical practice among a range of populations.

## Data Availability

The datasets used and/or analysed during the current study are available from the corresponding author on reasonable request.

## Abbreviations

Abd: Abduction
ACL: Anterior cruciate ligament
ACL_PT_: Anterior cruciate ligament physiotherapy-only treated group
ACL_R_: Anterior cruciate ligament reconstruction-treated group
Add: Adduction
ANOVA: Analysis of variance
BMI: Body mass index
CI: Confidence intervals
CTRL: Control group
Dom: CTRL dominant
ICC: Intraclass correlation coefficient
Inj: ACL-injured
KACL20-study: Knee injury - Anterior Cruciate Ligament after more than 20 years
LSI: Limb symmetry index
MU: Movement units
ND: CTRL non-dominant
NI: ACL non-injured
OA: Osteoarthritis
OLR: One-leg rise
SEM: Standard error of measurement
